# Polymorphisms in the Receptor Tyrosine Kinase *MERTK* Gene Are Associated with Multiple Sclerosis Susceptibility

**DOI:** 10.1371/journal.pone.0016964

**Published:** 2011-02-08

**Authors:** Gerry Z. M. Ma, Jim Stankovich, The Australia and New Zealand Multiple Sclerosis Genetics Consortium (ANZgene), Trevor J. Kilpatrick, Michele D. Binder, Judith Field

**Affiliations:** 1 Multiple Sclerosis Division, Florey Neuroscience Institutes, University of Melbourne, Melbourne, Victoria, Australia; 2 Centre for Neuroscience, University of Melbourne, Melbourne, Victoria, Australia; 3 Menzies Research Institute, University of Tasmania, Hobart, Tasmania, Australia; University of Texas M. D. Anderson Cancer Center, United States of America

## Abstract

Multiple sclerosis (MS) is a debilitating, chronic demyelinating disease of the central nervous system affecting over 2 million people worldwide. The TAM family of receptor tyrosine kinases (TYRO3, AXL and MERTK) have been implicated as important players during demyelination in both animal models of MS and in the human disease. We therefore conducted an association study to identify single nucleotide polymorphisms (SNPs) within genes encoding the TAM receptors and their ligands associated with MS. Analysis of genotype data from a genome-wide association study which consisted of 1618 MS cases and 3413 healthy controls conducted by the Australia and New Zealand Multiple Sclerosis Genetics Consortium (ANZgene) revealed several SNPs within the *MERTK* gene (Chromosome 2q14.1, Accession Number NG_011607.1) that showed suggestive association with MS. We therefore interrogated 28 SNPs in *MERTK* in an independent replication cohort of 1140 MS cases and 1140 healthy controls. We found 12 SNPs that replicated, with 7 SNPs showing *p*-values of less than 10^−5^ when the discovery and replication cohorts were combined. All 12 replicated SNPs were in strong linkage disequilibrium with each other. In combination, these data suggest the *MERTK* gene is a novel risk gene for MS susceptibility.

## Introduction

Multiple sclerosis (MS) is a chronic demyelinating and inflammatory disease of the central nervous system (CNS), affecting mainly individuals of European ancestry [1]. The disease is characterised by CNS demyelination, loss of oligodendrocytes, inflammation, as well as neurodegeneration. Susceptibility to MS is thought to involve a complex interplay of genetic and environmental factors, with the HLA-DRB1*1501-DQBI*602 (HLA-DR15) haplotype in the major histocompatibility complex (MHC) being the predominant genetic risk factor [2], [3]. Other genetic associations have been observed and replicated in *IL7R* [2], *IL2RA* [2], *CLEC16A* [4], [5], *CD226* [5], *CD6* [6], *IRF8* [6], *TNFRSF1A* [6], *STAT3* [7], *KIF21B* [8], *TMEM39A* [8] and *TYK2* [9].

The TAM receptors (TYRO3, AXL and MERTK) comprise a family of structurally related receptor tyrosine kinases that have two identified ligands: GAS6 and protein S [10], [11], [12]. TAM receptor signaling has been implicated in several biological processes, including cell survival and proliferation [13], [14], [15], [16], immune regulation [17], [18], [19] and phagocytosis of apoptotic cells [19], [20], [21]. As these are all key processes involved in demyelination, several recent studies have examined the role of these receptors in animal models of MS, as well as in the human disease.

Previous work in our laboratory examined the course of cuprizone-induced demyelination in mice lacking the TAM receptor ligand GAS6. Cuprizone is a neurotoxin that when incorporated into the feed of mice, induces specific and focal T cell-independent demyelination within the CNS, particularly in the corpus callosum [22], [23], [24]. Following 3 weeks of cuprizone-challenge we observed greater demyelination, which corresponded with increased oligodendrocyte loss and microglial activation in the absence of GAS6 compared with wild-type mice [25]. In a separate study, AXL receptor knockout mice displayed an overall reduction in myelination following 6 weeks of cuprizone-challenge, as well as a delay in microglial activation and the clearance of myelin debris and apoptotic cells [26]. The apparent differences between the phenotypes of the ligand and single receptor knockout mice during cuprizone-challenge highlights the complexity of TAM receptor signaling, but more importantly, these studies clearly show that loss of TAM receptor signaling is accompanied by increased demyelination in the cuprizone model.

The studies by Binder et al. [25] and Hoehn et al. [26] have focused on experimental animals, but a recent study has implicated the TAM receptors as important players in human MS as well. In a study of human MS lesions, levels of soluble forms of the TAM receptors, which can act as decoy receptors for the membrane-bound receptors, were compared in chronic silent MS lesions, chronic active MS lesions and healthy controls. It was found that levels of soluble AXL were higher in chronic silent lesions, while levels of soluble MERTK were higher in chronic active lesions compared with controls [27]. These elevated levels of soluble AXL and MERTK also correlated with low levels of GAS6 within the lesions [27], suggesting that loss of TAM receptor signaling may prolong MS lesion activity. Taken together, these studies implicate TAM receptor signaling as playing a key role in several processes affecting the outcome of demyelination in both animal models as well as human MS.

Given the aforementioned results, we hypothesized that polymorphisms in the TAM receptor or ligand genes would be associated with MS and thus also be involved in the aetiology of the disease. In a recent genome-wide association study (GWAS) conducted by the Australian and New Zealand Multiple Sclerosis Genetics Consortium (ANZgene) [28], several single nucleotide polymorphisms (SNPs) within the *MERTK* gene (Chromosome 2q14.1, Accession Number NG_011607.1) showed suggestive association with susceptibility to MS, while SNPs within the *TYRO3*, *AXL*, *GAS6* and *PROS1* genes did not show any suggestive associations. We therefore conducted a replication study with a candidate gene approach to validate this finding by genotyping 28 common SNPs within *MERTK* in an independent cohort of 1140 MS cases and 1140 healthy controls. In this study, we identify 12 replicated SNPs that are significantly associated with MS susceptibility, suggesting the *MERTK* gene as a novel risk gene involved in MS susceptibility.

## Methods

### Study subjects

The Melbourne Health Human Research Ethics Committee and the Australian Bone Marrow Donor Registry Ethics Committee granted approval for this research. Written consent was given by the subjects for their information to be stored in the study database and used for research.

Genotype data from a GWAS conducted by the ANZgene Consortium [28] was used as a discovery dataset to search the five TAM receptor and ligand genes for SNPs that showed suggestive association with MS susceptibility. Following quality control as outlined in the ANZgene GWAS [28], this dataset consisted of 1618 MS cases of European ancestry from Australia and New Zealand, and 3413 healthy controls of European ancestry from Australia, the UK and the US. All GWAS subjects were genotyped using Illumina arrays (Illumina, California, USA) [28].

A sample of 1140 MS cases and 1140 healthy controls from Australia and New Zealand were used as a replication cohort in this study (Table 1). Australian MS cases were volunteers recruited from Adelaide, Brisbane, Gold Coast, Hobart, Melbourne, Newcastle, Perth and Sydney. MS cases from New Zealand were recruited through a national prevalence survey. All cases had definite, clinically definite, or laboratory-supported definite MS according to the McDonald and Poser criteria, respectively. All controls were from the Australian Bone Marrow Donor Registry with no history of clinically isolated syndrome or MS at the time of collection. All samples were obtained from individuals of European ancestry from Australia and New Zealand.

**Table 1:** Replication sample data.

	MS patients (*n* = 1140)	Controls (*n* = 1140)
**Mean age of onset ± standard deviation** 1 **:**	33.85±10.14	N/A
**Sex:**		
Female	816	669
Male	247	450
Unknown	77	21
**MS disease course:**		N/A
RRMS	632	
SPMS	369	
PPMS	58	
Single	1	
Unknown	80	

MS: multiple sclerosis; N/A: not applicable; RRMS: relapsing-remitting multiple sclerosis; SPMS: secondary-progressive multiple sclerosis; PPMS: primary-progressive multiple sclerosis; Single: first demyelinating event.

1 Age information only available for 1069 MS patients and the mean is calculated as such.

### SNP selection

Of the top 7 directly genotyped SNPs in TAM receptor and ligand genes in the ANZgene GWAS, 6 were in the *MERTK* gene (Table S1). We therefore took a candidate gene approach focusing on *MERTK*. Using the total of 22 directly genotyped SNPs in the *MERTK* gene as proxies, non-genotyped SNPs that were tagged in the GWAS dataset were imputed using Beagle (see below). Based on this information, we selected 28 SNPs that showed suggestive association with MS to interrogate in the replication phase. These included the top 6 genotyped SNPs in the ANZgene GWAS, plus 22 imputed SNPs that showed suggestive association with MS.

### Genotyping

Subjects in the replication cohort were genotyped using the Sequenom MassARRAY system and iPLEX Gold chemistry under conditions recommended by the manufacturer (Sequenom, California, USA). Genomic DNA from all subjects was extracted as described in the ANZgene GWAS [28].

### Imputation

Imputation of tagged, un-genotyped SNPs in the GWAS cohort was performed with Beagle v3.2.0 (http://faculty.washington.edu/browning/beagle/beagle.html) [29] using the default settings. Analyses were performed to impute the most likely values of missing genotypes, and to calculate posterior genotype probabilities at all HapMap SNPs. To do this, data from unphased Hapmap phase II CEU (USA residents of northern and western European ancestry) individuals (release 24, build 36, forward strand) was used as a reference panel.

### Statistical analysis

For SNPs that were genotyped in both the GWAS and replication cohorts, association analysis was performed using the –assoc command in PLINK v1.07 (http://pngu.mgh.harvard.edu/~purcell/plink/) [30].

For SNPs that were imputed in either the GWAS or replication subjects, posterior allele dosages were used to perform the association analysis. The posterior allele dosage is twice the posterior genotype probability of the AA genotype plus the posterior genotype probability of the AB genotype (where A represents one of the alleles for the marker and B the other allele). Linear regression of allele dosages on case-control status was performed to test for association, with adjustment for sample group (discovery/replication) in the combined dataset.

### Linkage disequilibrium analysis

To investigate linkage disequilibrium (LD) between SNPs, data from Hapmap phase II CEU individuals (release 24, build 36, forward strand) was analysed using Haploview v4.2 (http://www.broadinstitute.org/haploview) [31].

### Hardy-Weinberg equilibrium analysis

Hardy-Weinberg equilibrium of SNPs was determined by using the –hardy command in PLINK v1.07. SNPs in significant Hardy-Weinberg disequilibrium (*p*<10^−7^) in control groups were excluded from further analysis.

## Results and Discussion

**Figure 1:**
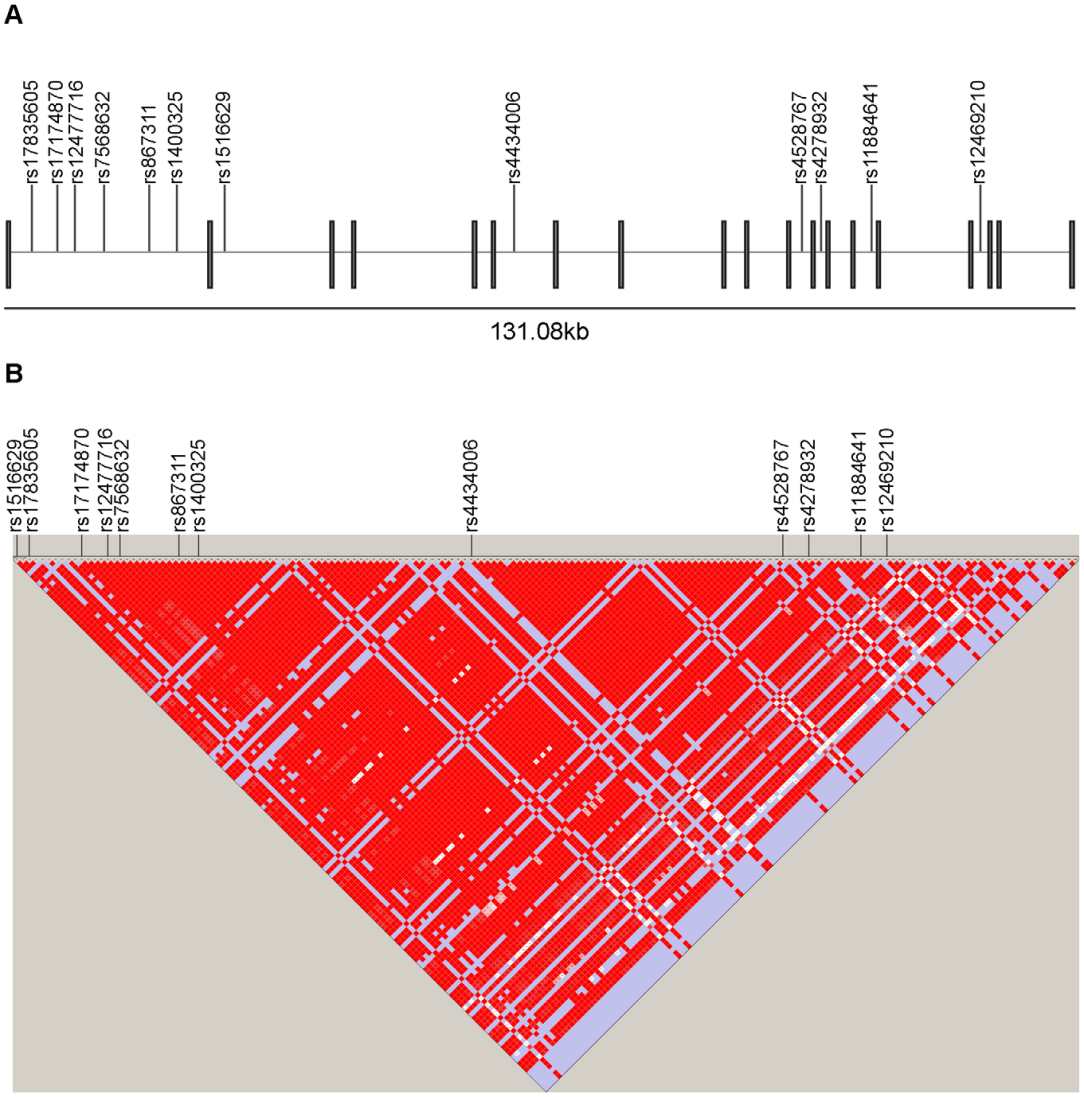
Location of SNPs within the *MERTK* gene. A. Schematic of the *MERTK* gene (Accession Number NG_011607.1, Chromosome 2: 12,656,056-112,787,138 forward strand). Black rectangles indicate the 19 exons of *MERTK* and grey lines show physical location of the 12 replicated SNPs. B. Linkage disequilibrium (LD) structure for all Hapmap phase II CEU SNPs (release 24, build 36, forward strand) in the *MERTK* gene, visualized using Haploview v4.2 (ref. 29). Lines point out the 12 replicated SNPs. Inter-SNP LD is represented by shaded squares, with red shading indicating strongest LD (*D*′ = 1), and white indicating weak or no LD between SNPs.

Our discovery dataset consisted of genotype data of 1618 MS cases and 3413 controls of European ancestry from a recent GWAS [28]. Of the top 7 directly genotyped SNPs in genes encoding TAM receptors and their ligands that showed suggestive *p*-values for association with MS in the discovery dataset, 6 were in the *MERTK* gene (Chromosome 2q14.1, Accession Number NG_011607.1) (Table S1). Based on association analysis of directly genotyped and imputed SNPs in the discovery dataset, we conducted a replication study with a candidate gene approach focusing on 28 SNPs within the *MERTK* gene. In our independent replication cohort, 12 of the 28 interrogated SNPs replicated, with association *p*-values of less than 0.05 and odds ratios going in the same direction as in the discovery dataset (Fig. 1A, Table 2). All 12 replicated SNPs had *p*-values of less than 10^-4^ when the discovery and replication cohorts were combined. Notable SNPs with combined *p*-values of less than 10^−5^ were rs867311, rs12477716, rs17835605, rs4278932, rs4528767, rs17174870 and rs1516629. Linkage disequilibrium analysis showed all 12 replicated SNPs to be in strong LD with each other, with *D*′ = 1 and *r* ^2^>0.95 for all pairs of SNPs (Fig. 1B).

**Table 2:** SNP associations across the *MERTK* gene in the discovery (GWAS), replication and combined datasets.

						GWAS						Replication						Combined			
SNP ID	Chr	Position^1^	Gene	Major Allele	Minor Allele	Gen/Imp	MAF cases	MAF controls	HWE *p*-value (controls)	*p*-value	OR	Gen/Imp	MAF cases	MAF controls	HWE *p*-value (controls)	*p*-value	OR	MAF cases	MAF controls	*p*-value^2^	OR
**rs867311**	**2**	**112,677,971**	***MERTK***	**G**	**T**	**Imp**	**0.223**	**0.249**	**0.783**	**5.37×10^−3^**	**0.87**	**Gen**	**0.224**	**0.263**	**0.017**	**2.41×10^−3^**	**0.81**	**0.223**	**0.251**	**5.41×10^−5^**	**0.86**
**rs12477716**	**2**	**112,668,645**	***MERTK***	**C**	**T**	**Imp**	**0.223**	**0.249**	**0.783**	**4.51×10^−3^**	**0.87**	**Gen**	**0.220**	**0.258**	**0.349**	**3.17×10^−3^**	**0.81**	**0.224**	**0.251**	**5.50×10^−5^**	**0.86**
**rs17835605**	**2**	**112,661,107**	***MERTK***	**C**	**T**	**Imp**	**0.223**	**0.249**	**0.783**	**4.51×10^−3^**	**0.87**	**Gen**	**0.226**	**0.263**	**0.053**	**4.49×10^−3^**	**0.82**	**0.222**	**0.251**	**5.96×10^−5^**	**0.85**
**rs4278932**	**2**	**112,761,311**	***MERTK***	**G**	**A**	**Imp**	**0.221**	**0.247**	**0.491**	**3.76×10^−3^**	**0.86**	**Gen**	**0.227**	**0.263**	**0.064**	**5.06×10^−3^**	**0.82**	**0.223**	**0.249**	**6.96×10^−5^**	**0.86**
**rs4528767**	**2**	**112,759,865**	***MERTK***	**G**	**C**	**Imp**	**0.221**	**0.247**	**0.491**	**3.76×10^−3^**	**0.86**	**Gen**	**0.225**	**0.261**	**4.24×10^−6^**	**5.38×10^−3^**	**0.82**	**0.225**	**0.251**	**5.91×10^−5^**	**0.87**
**rs17174870**	**2**	**112,665,202**	***MERTK***	**C**	**T**	**Imp**	**0.223**	**0.249**	**0.783**	**4.51×10^−3^**	**0.87**	**Gen**	**0.223**	**0.258**	**0.102**	**5.68× 10^−3^**	**0.82**	**0.224**	**0.252**	**7.76×10^−5^**	**0.86**
**rs1516629**	**2**	**112,688,680**	***MERTK***	**T**	**C**	**Imp**	**0.223**	**0.249**	**0.783**	**4.51×10^−3^**	**0.87**	**Gen**	**0.225**	**0.260**	**0.002**	**6.08×10^−3^**	**0.83**	**0.224**	**0.252**	**8.24×10^−5^**	**0.85**
**rs1400325**	**2**	**112,680,935**	***MERTK***	**G**	**A**	**Imp**	**0.223**	**0.249**	**0.783**	**5.37×10^−3^**	**0.87**	**Gen**	**0.227**	**0.258**	**0.212**	**0.01**	**0.84**	**0.224**	**0.251**	**2.25×10^−4^**	**0.86**
**rs4434006**	**2**	**112,728,812**	***MERTK***	**C**	**G**	**Imp**	**0.221**	**0.247**	**0.580**	**4.23×10^−3^**	**0.87**	**Gen**	**0.226**	**0.258**	**0.160**	**0.01**	**0.84**	**0.225**	**0.251**	**1.79×10^−4^**	**0.86**
**rs11884641**	**2**	**112,767,407**	***MERTK***	**A**	**G**	**Gen**	**0.221**	**0.247**	**0.491**	**3.80×10^−3^**	**0.86**	**Gen**	**0.231**	**0.261**	**0.163**	**0.02**	**0.85**	**0.142**	**0.196**	**1.79×10^−4^**	**0.68**
**rs7568632**	**2**	**112,669,951**	***MERTK***	**A**	**G**	**Imp**	**0.223**	**0.249**	**0.783**	**4.51×10^−3^**	**0.87**	**Gen**	**0.223**	**0.252**	**0.525**	**0.02**	**0.85**	**0.223**	**0.251**	**1.89×10^−4^**	**0.86**
**rs12469210**	**2**	**112,768,992**	***MERTK***	**G**	**A**	**Imp**	**0.222**	**0.248**	**0.436**	**3.96×10^−3^**	**0.86**	**Gen**	**0.226**	**0.252**	**0.475**	**0.04**	**0.87**	**0.223**	**0.249**	**4.06×10^−4^**	**0.87**
rs1516640	2	112,674,859	*MERTK*	G	A	Gen	0.479	0.445	0.755	1.57×10^−3^	1.15	Gen	0.480	0.456	0.061	0.10	1.10	0.461	0.459	4.45×10^−4^	1.01
rs4848901	2	112,710,829	*MERTK*	G	A	Gen	0.476	0.443	0.627	1.88×10^−3^	1.14	Gen	0.467	0.447	0.069	0.17	1.09	0.458	0.453	7.91×10^−4^	1.02
rs13419523	2	112,781,918	*MERTK*	A	G	Gen	0.086	0.069	0.894	3.32×10^−3^	1.26	Gen	0.077	0.066	0.224	0.15	1.18	0.078	0.070	1.27×10^−3^	1.13
rs6730521	2	112,783,958	*MERTK*	A	G	Gen	0.221	0.247	0.462	4.20×10^−3^	0.86	Gen	0.217	0.232	1.000	0.24	0.92	0.235	0.234	1.83×10^−3^	1.00
rs3811632	2	112,754,829	*MERTK*	C	A	Gen	0.272	0.293	0.934	0.03	0.90	Gen	0.264	0.273	0.082	0.50	0.96	0.283	0.279	0.03	1.02

SNPs in bold are the 12 SNPs that show association in both the discovery and replication cohorts. Un-bolded SNPs are SNPs genotypes in the discovery dataset that showed suggestive association, but failed to replicate. SNP, single nucleotide polymorphism; GWAS, genome-wide association study; Chr, Chromosome; Gen, genotyped; Imp, imputed; MAF, minor allele frequency; HWE, Hardy-Weinberg equilibrium; OR, odds ratio.

1 SNP positions are from the Ensembl Genome Browser (http://www.ensembl.org/index.html, October 2010. Acc: 7027).

2 Analysis of the combined dataset was adjusted for sample group (discovery/replication).

We included the top 6 genotyped SNPs from the discovery dataset with *p*-values of less than 0.05 in the 28 SNPs that were genotyped in our replication cohort. Of these SNPs, only rs11884641 replicated, with the other 5 showing *p*-values of greater than 0.05 in the replication cohort (Table 2).

Although the SNPs interrogated in this study do not reach the level of genome-wide significance (*p*<5×10^−8^), our study nevertheless identifies 12 SNPs that replicate in two independent cohorts of MS cases and controls, and that reach a level of significance required for a candidate gene approach. This provides strong evidence that the *MERTK* gene is associated with MS susceptibility. The 12 replicated SNPs are all common variants; although all are intronic and in strong LD with each other, they may be tagging potentially rare causal variants within coding regions of the gene. Fine mapping of the *MERTK* gene and interrogation of different populations with larger sample sizes may aid in determining the exact causal variants. As MS is a genetically complex disease, the *MERTK* gene may also represent one of many disease loci that confer susceptibility to MS when present in combination.

The TAM receptors have been shown to be major players in regulation of the immune response [17], [18], [19]. Triple TAM receptor mutants develop widespread autoimmunity, with increased circulating levels of autoantibodies [17]. The autoimmunity phenotype showed a gene dosage effect, with less severe autoimmunity in mice deficient for two TAM receptors, and an even milder, although still pronounced effect in mice deficient for just one TAM receptor [17]. The role of MERTK in regulating the immune response has been extensively examined, especially in relation to its role in the phagocytosis of apoptotic cells. It has been shown that MERTK knockout mice have macrophages deficient in their ability to clear apoptotic cells, leading to the development of a progressive systemic lupus erythematosus-like phenotype [20], [32]. In the immune system, MERTK has also been implicated in regulation of dendritic cell activation [33], T cell selection and tolerance [34], [35], and B cell homeostasis and tolerance [36], [37].

Multiple sclerosis has often been classified as an autoimmune disease, and the obvious inflammatory response observed in the disease strongly supports this hypothesis. In light of previous studies implicating the TAM receptors in regulating both demyelination and autoimmune responses in both experimental animals and human MS lesions (for review, see ref. [38]), the present study provides strong evidence that *MERTK* represents a genuine MS susceptibility gene.

In conclusion, this candidate gene study has identified an association of MS with 12 SNPs in the *MERTK* gene that replicated in two independent cohorts of MS cases and controls, an association that is particularly compelling given the previous studies implicating TAM receptor signalling in demyelination and autoimmunity. Further fine mapping studies will be required to determine the causal variant or variants.

## Supporting Information

Table S1 SNP associations of all directly genotyped TAM receptor and ligand genes in the top 300,000 SNPs of the discovery (GWAS) dataset.
(DOC)

## References

[1] Compston A, Coles A (2008). Multiple sclerosis.. Lancet.

[2] Hafler DA, Compston A, Sawcer S, Lander ES, Daly MJ (2007). Risk alleles for multiple sclerosis identified by a genomewide study.. N Engl J Med.

[3] Masterman T, Ligers A, Olsson T, Andersson M, Olerup O (2000). HLA-DR15 is associated with lower age at onset in multiple sclerosis.. Ann Neurol.

[4] Rubio JP, Stankovich J, Field J, Tubridy N, Marriott M (2008). Replication of KIAA0350, IL2RA, RPL5 and CD58 as multiple sclerosis susceptibility genes in Australians.. Genes Immun.

[5] International Multiple Sclerosis Genetics Consortium (2009). The expanding genetic overlap between multiple sclerosis and type I diabetes.. Genes Immun.

[6] De Jager PL, Jia X, Wang J, de Bakker PI, Ottoboni L (2009). Meta-analysis of genome scans and replication identify CD6, IRF8 and TNFRSF1A as new multiple sclerosis susceptibility loci.. Nat Genet.

[7] Jakkula E, Leppa V, Sulonen AM, Varilo T, Kallio S (2010). Genome-wide association study in a high-risk isolate for multiple sclerosis reveals associated variants in STAT3 gene.. Am J Hum Genet.

[8] International Multiple Sclerosis Genetics Consortium (2010). Comprehensive follow-up of the first genome-wide association study of multiple sclerosis identifies KIF21B and TMEM39A as susceptibility loci.. Hum Mol Genet.

[9] Ban M, Goris A, Lorentzen AR, Baker A, Mihalova T (2009). Replication analysis identifies TYK2 as a multiple sclerosis susceptibility factor.. Eur J Hum Genet.

[10] Godowski PJ, Mark MR, Chen J, Sadick MD, Raab H (1995). Reevaluation of the roles of protein S and Gas6 as ligands for the receptor tyrosine kinase Rse/Tyro 3.. Cell.

[11] Stitt TN, Conn G, Gore M, Lai C, Bruno J (1995). The anticoagulation factor protein S and its relative, Gas6, are ligands for the Tyro 3/Axl family of receptor tyrosine kinases.. Cell.

[12] Nagata K, Ohashi K, Nakano T, Arita H, Zong C (1996). Identification of the product of growth arrest-specific gene 6 as a common ligand for Axl, Sky, and Mer receptor tyrosine kinases.. J Biol Chem.

[13] Yagami T, Ueda K, Asakura K, Sakaeda T, Nakazato H (2002). Gas6 rescues cortical neurons from amyloid beta protein-induced apoptosis.. Neuropharmacology.

[14] Shankar SL, O'Guin K, Cammer M, McMorris FA, Stitt TN (2003). The growth arrest-specific gene product Gas6 promotes the survival of human oligodendrocytes via a phosphatidylinositol 3-kinase-dependent pathway.. J Neurosci.

[15] Shankar SL, O'Guin K, Kim M, Varnum B, Lemke G (2006). Gas6/Axl signaling activates the phosphatidylinositol 3-kinase/Akt1 survival pathway to protect oligodendrocytes from tumor necrosis factor alpha-induced apoptosis.. J Neurosci.

[16] Li R, Chen J, Hammonds G, Phillips H, Armanini M (1996). Identification of Gas6 as a growth factor for human Schwann cells.. J Neurosci.

[17] Lu Q, Lemke G (2001). Homeostatic regulation of the immune system by receptor tyrosine kinases of the Tyro 3 family.. Science.

[18] Rothlin CV, Ghosh S, Zuniga EI, Oldstone MB, Lemke G (2007). TAM receptors are pleiotropic inhibitors of the innate immune response.. Cell.

[19] Lemke G, Rothlin CV (2008). Immunobiology of the TAM receptors.. Nat Rev Immunol.

[20] Scott RS, McMahon EJ, Pop SM, Reap EA, Caricchio R (2001). Phagocytosis and clearance of apoptotic cells is mediated by MER.. Nature.

[21] Anderson HA, Maylock CA, Williams JA, Paweletz CP, Shu H (2003). Serum-derived protein S binds to phosphatidylserine and stimulates the phagocytosis of apoptotic cells.. Nat Immunol.

[22] Hiremath MM, Saito Y, Knapp GW, Ting JP, Suzuki K (1998). Microglial/macrophage accumulation during cuprizone-induced demyelination in C57BL/6 mice.. J Neuroimmunol.

[23] Matsushima GK, Morell P (2001). The neurotoxicant, cuprizone, as a model to study demyelination and remyelination in the central nervous system.. Brain Pathol.

[24] Hiremath MM, Chen VS, Suzuki K, Ting JP, Matsushima GK (2008). MHC class II exacerbates demyelination in vivo independently of T cells.. J Neuroimmunol.

[25] Binder MD, Cate HS, Prieto AL, Kemper D, Butzkueven H (2008). Gas6 deficiency increases oligodendrocyte loss and microglial activation in response to cuprizone-induced demyelination.. J Neurosci.

[26] Hoehn HJ, Kress Y, Sohn A, Brosnan CF, Bourdon S (2008). Axl-/- mice have delayed recovery and prolonged axonal damage following cuprizone toxicity.. Brain Res.

[27] Weinger JG, Omari KM, Marsden K, Raine CS, Shafit-Zagardo B (2009). Up-regulation of soluble Axl and Mer receptor tyrosine kinases negatively correlates with Gas6 in established multiple sclerosis lesions.. Am J Pathol.

[28] Australia and New Zealand Multiple Sclerosis Genetics Consortium (2009). Genome-wide association study identifies new multiple sclerosis susceptibility loci on chromosomes 12 and 20.. Nat Genet.

[29] Browning BL, Browning SR (2009). A unified approach to genotype imputation and haplotype-phase inference for large data sets of trios and unrelated individuals.. Am J Hum Genet.

[30] Purcell S, Neale B, Todd-Brown K, Thomas L, Ferreira MA (2007). PLINK: a tool set for whole-genome association and population-based linkage analyses.. Am J Hum Genet.

[31] Barrett JC, Fry B, Maller J, Daly MJ (2005). Haploview: analysis and visualization of LD and haplotype maps.. Bioinformatics.

[32] Cohen PL, Caricchio R, Abraham V, Camenisch TD, Jennette JC (2002). Delayed apoptotic cell clearance and lupus-like autoimmunity in mice lacking the c-mer membrane tyrosine kinase.. J Exp Med.

[33] Sen P, Wallet MA, Yi Z, Huang Y, Henderson M (2007). Apoptotic cells induce Mer tyrosine kinase-dependent blockade of NF-kappaB activation in dendritic cells.. Blood.

[34] Wallet MA, Flores RR, Wang Y, Yi Z, Kroger CJ (2009). MerTK regulates thymic selection of autoreactive T cells.. Proc Natl Acad Sci U S A.

[35] Wallet MA, Sen P, Flores RR, Wang Y, Yi Z (2008). MerTK is required for apoptotic cell-induced T cell tolerance.. J Exp Med.

[36] Shao WH, Eisenberg RA, Cohen PL (2008). The Mer receptor tyrosine kinase is required for the loss of B cell tolerance in the chronic graft-versus-host disease model of systemic lupus erythematosus.. J Immunol.

[37] Shao WH, Kuan AP, Wang C, Abraham V, Waldman MA (2010). Disrupted Mer receptor tyrosine kinase expression leads to enhanced MZ B-cell responses.. J Autoimmun.

[38] Binder MD, Kilpatrick TJ (2009). TAM receptor signalling and demyelination.. Neurosignals.

