# Supplementary material for: Polymorphisms in the Receptor Tyrosine Kinase MERTK Gene Are Associated with Multiple Sclerosis Susceptibility
Source: PLoS One. 2011 Feb 8;6(2):e16964. doi: 10.1371/journal.pone.0016964 (PMC3035668; doi:10.1371/journal.pone.0016964)
Supplement: Table S1 — SNP associations of all directly genotyped TAM receptor and ligand genes in the top 300,000 SNPs of the discovery (GWAS) dataset. (DOC) [file pone.0016964.s001.doc]

**Table S1. SNP associations of all directly genotyped TAM receptor and ligand genes in the top 300,000 SNPs of the discovery (GWAS) dataset.**

| **SNP** | **Chromosome** | **Gene** | ***p*-value** |
| --- | --- | --- | --- |
| rs1516640 | 2 | *MERTK* | 1.43 × 10^-3^ |
| rs4848901 | 2 | *MERTK* | 1.69 × 10^-3^ |
| rs11884641 | 2 | *MERTK* | 3.67 × 10^-3^ |
| rs13419523 | 2 | *MERTK* | 3.75 × 10^-3^ |
| rs6730521 | 2 | *MERTK* | 4.02 × 10^-3^ |
| rs12259 | 15 | *TYRO3* | 9.41 × 10^-3^ |
| rs3811632 | 2 | *MERTK* | 0.03 |
| rs6602910 | 13 | *GAS6* | 0.06 |
| rs11123073 | 2 | *MERTK* | 0.11 |
| rs7604639 | 2 | *MERTK* | 0.11 |
| rs9604573 | 13 | *GAS6* | 0.12 |
| rs4535048 | 2 | *MERTK* | 0.14 |
| rs9944249 | 15 | *TYRO3* | 0.16 |
| rs8178591 | 3 | *PROS1* | 0.28 |
| rs8000868 | 13 | *GAS6* | 0.28 |
| rs7644769 | 3 | *PROS1* | 0.31 |
| rs8178610 | 3 | *PROS1* | 0.31 |
| rs8178607 | 3 | *PROS1* | 0.31 |
| rs11842558 | 13 | *GAS6* | 0.32 |
| rs7996080 | 13 | *GAS6* | 0.39 |
| rs13027171 | 2 | *MERTK* | 0.48 |
| rs4803448 | 19 | *AXL* | 0.49 |
| rs6736093 | 2 | *MERTK* | 0.51 |
| rs10180086 | 2 | *MERTK* | 0.54 |
| rs11690295 | 2 | *MERTK* | 0.54 |
| rs3811633 | 2 | *MERTK* | 0.56 |
| rs3811634 | 2 | *MERTK* | 0.58 |
| rs2304234 | 19 | *AXL* | 0.62 |
| rs13062355 | 3 | *PROS1* | 0.69 |
| rs2304232 | 19 | *AXL* | 0.74 |
| rs11687619 | 2 | *MERTK* | 0.77 |
| rs12459996 | 19 | *AXL* | 0.81 |
| rs2271546 | 19 | *AXL* | 0.87 |
